# Knowledge and practices about health among *Quilombola* men: contributions to health care

**DOI:** 10.1590/0034-7167-2023-0138

**Published:** 2023-12-08

**Authors:** Felipe Valino dos Santos, Ivaneide Leal Ataíde Rodrigues, Laura Maria Vidal Nogueira, Erlon Gabriel Rego de Andrade, Aloma Sena Soares, Élida Fernanda Rêgo de Andrade

**Affiliations:** I Universidade do Estado do Pará. Belém, Pará, Brazil

**Keywords:** *Quilombola* Communities, Health of Ethnic Minorities, Men’s Health, Health Behavior, Vulnerable Populations, Quilombola, Salud de las Minorías Étnicas, Salud del Hombre, Conductas Relacionadas con la Salud, Poblaciones Vulnerables, Quilombolas, Saúde das Minorias Étnicas, Saúde do Homem, Comportamentos Relacionados com a Saúde, Populações Vulneráveis

## Abstract

**Objective:**

to analyze health knowledge and practices among *Quilombola* men.

**Methods:**

a qualitative, descriptive study, carried out with 40 men from two *Quilombola* communities in Santa Izabel do Pará, state of Pará, Brazil. Individual interviews were carried out using a semi-structured script. Text *corpus* was subjected to analysis with *Interface de R pour les Analyses Multidimensionnelles de Textes et de Questionnaires* 0.6, alpha 3, through Descending Hierarchical Classification.

**Results:**

among participants, eight (20.00%) were aged 55 to 59 years. 382 text segments were identified, with 299 (78.27%) being used, generating five lexical classes, which made up two *subcorpora*. The classes were organized into four thematic axes, covering knowledge about health and practices to prevent and solve health problems.

**Final considerations:**

men highlighted popular/traditional wisdom permeated by biomedical knowledge, translating their understanding of how to act to remain or become healthy.

## RESUMO

**Objetivo:**

analisar os saberes e as práticas sobre saúde entre homens quilombolas.

**Métodos:**

estudo qualitativo, descritivo, realizado com 40 homens de duas comunidades quilombolas de Santa Izabel do Pará, estado do Pará, Brasil. Realizaram-se entrevistas individuais, utilizando roteiro semiestruturado. O *corpus* textual foi submetido à análise com o *software Interface de R pour les Analyses Multidimensionnelles de Textes et de Questionnaires* 0.6, *alpha* 3, por meio da Classificação Hierárquica Descendente.

**Resultados:**

entre os participantes, oito (20,00%) estavam na faixa etária de 55 a 59 anos. Identificaram-se 382 segmentos de textos, com aproveitamento de 299 (78,27%), gerando cinco classes lexicais, que compuseram dois *subcorpora*. As classes foram organizadas em quatro eixos temáticos, abordando os saberes sobre saúde e as práticas para prevenir e resolver problemas de saúde.

**Considerações finais:**

os homens destacaram a sabedoria popular/tradicional permeada por conhecimentos biomédicos, traduzindo suas compreensões sobre como agir para permanecerem ou se tornarem saudáveis.

## RESUMEN

**Objetivo:**

analizar conocimientos y prácticas de salud entre hombres *quilombolas*.

**Métodos:**

estudio cualitativo, descriptivo, realizado con 40 hombres de dos comunidades *quilombolas* de Santa Izabel do Pará, estado de Pará, Brasil. Las entrevistas individuales se realizaron mediante un guión semiestructurado. El *corpus* textual fue sometido a análisis con *Interface de R pour les Analyses Multidimensionnelles de Textes et de Questionnaires* 0.6, *alpha* 3, mediante Clasificación Jerárquica Descendente.

**Resultados:**

entre los participantes, ocho (20,00%) tenían entre 55 y 59 años. Se identificaron 382 segmentos textuales, de los cuales se utilizaron 299 (78,27%), generándose cinco clases léxicas, que conformaron dos *subcorpora*. Las clases se organizaron en cuatro ejes temáticos, abarcando conocimientos sobre salud y prácticas para prevenir y resolver problemas de salud.

**Consideraciones finales:**

los hombres resaltaron la sabiduría popular/tradicional impregnada de conocimientos biomédicos, traduciendo su comprensión de cómo actuar para permanecer o volverse saludables.

## INTRODUCTION

In general, men have been used to avoiding contact with health services, taking pride in the idea that they are invulnerable. Averse to prevention and self-care, it is common for them to delay the search for professional care, often resulting in the worsening of clinical cases and greater expenses for themselves and the health system, which is forced to intervene in the most advanced stages of diseases^(1)^.

A significant portion of non-adherence to health care measures arises from cultural variables, in addition to the fact that gender stereotypes, rooted for centuries in Brazilian patriarchal culture, enhance practices based on beliefs and values about what it means to be male. Thus, men believe they are invulnerable, contributing to them taking less care of themselves and exposing themselves to risk situations on a daily basis^(2, 3)^.

From this perspective, the social heterogeneity of the possibilities of being a man must be considered, a context in which masculinities are historically constructed, as a process in constant transformation. This aspect is fundamental to promoting equity in men’s health care, which must be considered and valued according to differences in age, socioeconomic and ethnic-racial condition, urban or rural place of residence, prison situation, physical and/or intellectual disability, sexual orientations and non-hegemonic gender identities^(4, 5, 6)^.

In view of this, the male population is inserted in different social arrangements, presenting many complexities, which is why it is necessary to understand the reality of each human group and its influence on populations’ lives and social organization. Likewise, it is essential to recognize the social determinants that result in men’s vulnerable condition, considering that stereotypical representations of masculinity can compromise access to necessary care^(7, 8)^.

The analysis of subjects’ sociocultural environments contributes to understanding their life and health context, in addition to guiding service actions to meet individual and collective needs. Some communities are inserted in scenarios that make them even more vulnerable, such as traditional peoples, such as *Quilombola* (*Quilombola* is a common designation for slaves who were refugees in *quilombos*, or descendants of black slaves, and they live in *quilombos*) communities, who historically resist and survive limitations of different natures. All life and work relationships of these people are closely linked to the natural and cultural environment in which they reside^(8, 9)^.

Often, in health services’ daily routine, there is a lack of recognition of the particularities inherent to men’s health, especially *Quilombola*, to promote self-care. This reality intensifies their vulnerabilities, as they are part of contexts in which self-care practice is not valued and/or encouraged, due to socially constructed concepts and imaginaries of masculinity^(8)^.

Thus, there are inconsistencies in health habits among men, delaying the search for formal care until the moment they are unable to take care of themselves, due to worsening clinical conditions. In this scenario, it is essential to understand the specificities of *Quilombola* men’s practices, aiming to reduce inequities, in addition to producing and mobilizing new self-care management strategies, culminating in comprehensive and equitable health promotion^(8)^.

*Quilombola* communities’ living conditions strongly demonstrate greater precariousness, compared to those of the urban population. In rural areas, there are difficulties in accessing the health system, mainly related to the behavior adopted among users, especially males, as the participation of *Quilombola* men in health services is still scarce. At least partially, this is based on the fact that these men dedicate most of their time to work activities, taking responsibility for supporting their families, limiting the search for Primary Health Care (PHC) services, in order to promote health and prevent illness^(9, 10)^.

## OBJECTIVE

To analyze health knowledge and practices among *Quilombola* men.

## METHODS

### Ethical aspects

Resolution 466/2012 of the Brazilian National Health Council was complied with. The project was authorized by the *Secretaria de Saúde de Santa Izabel do Pará* (SESAU - Health Department of Santa Izabel do Pará) and approved by the Research Ethics Committee of the *Universidade do Estado do Pará* Undergraduate Nursing Course. All participants were presented with the Informed Consent Form (ICF), and the confidentiality of their identities was assured using an alphanumeric code, with the letter P, for the word “participant”, followed by the order number of the interviews.

### Study design

This is a descriptive study, with a qualitative approach, guided by COnsolidated criteria for REporting Qualitative research (COREQ)^(11)^.

### Study setting and data source

The research was carried out in two *Quilombola* communities in the municipality of Santa Izabel do Pará, state of Pará, Brazil: Boa Vista do Itá and Santa Luzia do Macapazinho. Located 36 km from the capital, Belém, this municipality is made up of three districts, Santa Izabel (municipal headquarters), Americano and Caraparu, in addition to several small towns.

Boa Vista do Itá is a community in the Caraparu district, approximately 60 km from Belém. In turn, the Santa Luzia do Macapazinho community is located approximately 15 km from the municipal headquarters. Both have access to health services through the Family Health Strategy (FHS) Conceição do Itá, which assists the entire region of the Caraparu district^(8, 12)^.

A total of 40 men participated: 17 from the Boa Vista do Itá community and 23 from the Santa Luzia do Macapazinho community. These numbers corresponded, respectively, to 42.50% (n=17/40) and 57.50% (n=23/40) of the male population of these communities, registered with FHS Conceição do Itá. Adult men residing in these communities, aged 25 to 59 years, were included. An individual within this range was considered an adult, according to the age classification provided for in the Brazilian National Policy for Comprehensive Health Care for Men (PNAISH - *Política Nacional de Atenção Integral à Saúde do Homem*)^(13)^. It was decided to exclude those who were not found in their homes after three attempts in the period scheduled to collect the data.

### Data collection and organization

Approaching the communities, the main researcher visited FHS Conceição do Itá to learn about the health services, talk about the project with the professionals who worked there and carry out a survey of the male population eligible to participate in the study.

Participants were approached on the premises of the health unit where FHS activities were carried out. They were invited, individually, by the researcher, when attending routine consultations and/or during other activities in the unit. Individual interviews were carried out with those who agreed to participate in a room provided by the health team or at home, on a previously agreed date and time. The interviews were recorded in MP3 format, with formal consent, and carried out ensuring participant comfort and privacy, seeking an environment conducive to dialogue.

In the interviews, a semi-structured script was used, prepared by the researchers and composed of two parts: the first with 13 questions referring to participant sociodemographic profile; and the second with five subjective questions to understand the object of study. These questions investigated individual understanding of health, the importance they attributed to the idea of being healthy and the reasons that led them to consider this condition, habits and practices that they considered conducive to maintaining health, what they did to solve health problems, where and how they learned these practices, and how they could influence health and well-being. Data collection took place from July to December 2019.

### Data analysis

The interviews were transcribed to create the *corpus*, which was subjected to lexical analysis with *Interface de R pour les Analyzes Multidimensionnelles de Textes et de Questionnaires* (IRaMuTeQ), version 0.6, alpha 3. Developed by Pierre Ratinaud, IRaMuTeQ allows processing and analysis statistically text *corpus*, guaranteeing reliability to the research^(14)^.

Among the analysis techniques available in the software, we chose the Descending Hierarchical Classification (DHC)^(14)^. Valuing clarity in the presentation of results, with the aim of expanding the reader’s understanding, the lexical classes generated by DHC were organized into thematic axes, highlighting representative words and some emblematic excerpts to demonstrate and *Quilombola* men’s subjectivities.

With their respective classes, thematic axes were interpreted and discussed in light of relevant scientific literature and guidelines and recommendations available in official documents from the Ministry of Health. In turn, sociodemographic profile data were analyzed descriptively to identify absolute values and respective percentages.

## RESULTS

Among the participants, eight (20.00%) were aged between 55 and 59; 17 (42.50%) were Catholic; 19 (47.50%) reported incomplete elementary school; 12 (30.00%) were farmers and peasants or traders; 29 (72.50%) declared a personal monthly income less than or equal to one minimum wage, and 22 (55.00%) declared a family income of one to two minimum wages; 23 (57.50%) lived in brick houses; 36 (90.00%) reported having an intra-domestic bathroom; 27 (67.50%) used water from a shallow well; 10 (25.00%) used landfills for organic waste; and seven (17.50%) reused recyclable waste.

**Figure 1:**
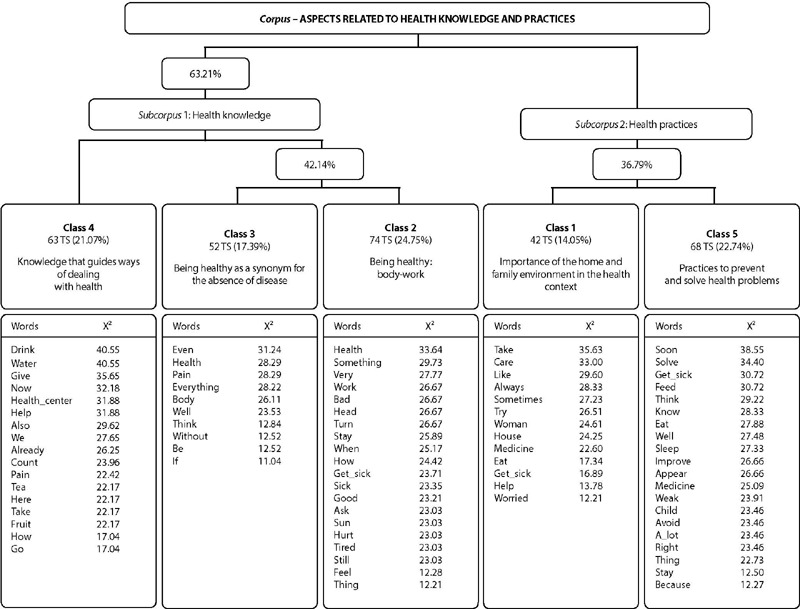
Dendrogram of descending hierarchical classification, Belém, Pará, Brazil, 2019

The *corpus* called “Aspects related to health knowledge and practices” consisted of 40 texts, broken down into 382 text segments (TS), of which 299 (78.27%) were used, generating five classes through DHC, as illustrated in Figure 1. Classes 4, 3 and 2 made up the first *subcorpus* called “Health knowledge”, and classes 1 and 5, the second *subcorpus* called “Health practices”.

The classes were organized into four thematic axes, respectively: “Knowledge that guides ways of dealing with health”, represented by class 4; “Being healthy as a synonym for the absence of disease and body-work”, represented by classes 3 and 2; “Importance of the home and family environment in the health context”, for class 1; and “Practices to prevent and solve health problems”, represented by class 5.

It should be noted that, to organize the thematic axes, the authors considered the classes in this order (4, 3, 2, 1 and 5), due to *corpus* processing by IRaMuTeQ, which generated this partition logic (Figure 1). Below are the axes and their respective classes.

### Thematic axis 1 – Knowledge that guides ways of dealing with health (class 4)

Composed of 63 TS (21.07% of the *corpus*), this class presents 17 representative words with greater strength (chi-square test – X^2^), building the idea of how *Quilombola* men act in accordance with what they learned about health at some point of their lives. Thus, it demonstrates the knowledge and learning acquired among men to maintain health and how they behave to promote physical and emotional well-being based on this knowledge.

Words “pain” (X^2^=22.42), “health_center” (X^2^=31.88), “tea” (X^2^=22.17) and “drink” (X^2^=40.55) illustrate a context of actions that portray how health is closely linked to disease treatment or prevention. TS present ways of producing health that dialogue with common sense, which is why they learn in the family environment from a very early age, in intimate relationships with ancestral generations.

TS also demonstrate how culture is shaped by this popular knowledge and learning, as many participants described using fruits, roots and plants, in the form of compresses or drinking tea, to treat symptoms such as headaches and back pain and combat to respiratory diseases. Furthermore, the adoption of care arising from biomedical knowledge was evidenced, through health services, with medicines available at the health unit or purchased commercially at pharmacies, during trips to the urban center of the region:

*We try to resolve things as we can, our mother makes tea to cure the flu, makes us chew leaves with honey. When it doesn’t work, I go to the pharmacy at the health center and ask the nurse for some medicine.* (P1)

### Thematic axis 2 – Being healthy as a synonym for the absence of disease and body-work (classes 3 and 2)

Together, classes 3 and 2 elucidate participants’ understanding of the set of factors that demonstrate health and physical or mental well-being ideas. They present an understanding of what it means to be healthy, with class 2 differing solely by treating work as a dimension that occurred with greater force in statements. From this perspective, the analysis of the two occurred separately, but in a complementary way, as they are closely related and consolidated.

Built with 52 TS (17.39% of the *corpus*), class 3 expresses knowledge about health linked to perceptions of physical body, emotional state and how this interferes with men’s routine. Representative words, such as “health” (X^2^=28.29), “pain” (X^2^=28.29), “body” (X^2^=26.11), “everything” (X^2^=28.22), “well” (X^2^=23.53) and “without” (X^2^=12.52), construct the concept of a healthy being in the conception of the absence of diseases or signs and symptoms.

The majority of statements (n=27; 67.50%) related “health” (X^2^=28.29) with “without” (X^2^=12.52), “pain” (X^2^=28.29) and “body” (X^2^=26.11), and engendered the understanding that health and illness are opposite phenomena and do not cohabit in the same body. In this regard, physical pain is indicative of not being well or healthy. Furthermore, participants related health to a temporary state of bodily stability, such as the absence of pain or other symptoms, even with the possibility of living with chronic diseases:

*Health is when my body is not in pain and I am not sick. Health comes when I feel good about myself and can do whatever I want. It is important for us to be able to live well,* [...] *without our health, we do nothing!* (P6)

In turn, class 2 was constructed with 74 TS (24.75% of the *corpus*), appearing as the largest class in the *corpus*. It demonstrates how health is characterized by the ability to perform work activities, presenting reflections that elucidate how these daily habits and the work environment itself can contribute to promoting or treating health and balance of body and mind.

Approaching representative words, such as “health” (X^2^=33.64), “something” (X^2^=29.73), “very” (X^2^=27.77), “good” (X^2^=23.21), “work” (X^2^=26.67) and “sun” (X^2^=23.03), defines the state of health, associating it with what comes closest to participants’ reality. Therefore, understanding what it means to be healthy, based on work experiences, seems to be one of the simplest ways to talk about health.

When asked about the importance of work in the concept of health, statements converged on a process of dignification as a means of obtaining material goods and good memories. Many statements corresponded to men who had a greater relationship with land, through agricultural activities. In this sense, land is considered a generator of income, food, household utilities, well-being and survival, and keeping it preserved is an essential task:

*Health is when we are well, happy, when we can work without complaining about pain or suffering. Yes, that is health. If I work well, I am healthy.* (P12)

### Thematic axis 3 – Importance of the home and family environment in the health context (class 1)

Created with 42 TS (14.05% of the *corpus*), this class presents representative words, such as “care” (X^2^=33.00), “take” (X^2^=35.63), “ e a t ” ( X ^2^=17.34), “get_sick” (X^2^=16.89) and “help” (X^2^=13.78), which demonstrate actions that refer to the close relationship with health practices, illustrating what *Quilombola* men do when they associate health and well-being with their practices or with the attention they receive from another person.

Mostly (n=34; 85.00%), participants reported that their practices involve two dimensions: family nucleus, especially represented by the mention of word “woman” (X^2^=24.61) as a social actor responsible for caring for men and with children; and domestic environment, represented by word “home” (X^2^=24.25), which appears as an agent that mobilizes health maintenance, illustrating the importance of a clean and safe environment that provides health and well-being, with access to basic services, such as sanitation, adequate disposal of solid waste and access to drinking water. From this perspective, keeping the house clean and organized makes men feel good and capable of taking care of themselves:

*I’m very lucky to have a house like mine, with this big plot of land, where I can work, rest, spend time with my family. I think that if I hadn’t been able to have a house like that, I would have gotten sicker.* (P36)

### Thematic axis 4 – Practices to prevent and solve health problems (class 5)

Composed of 68 TS (22.74% of the *corpus*), this class highlights care through interventions that demonstrate how health is energized and reconstituted with actions to treat, maintain or restore health and well-being.

Predominantly (n=33; 82.50%), the statements showed that men try to solve their problems using medicine, eating well and adopting other practices that they consider appropriate. The search for a health unit for general prevention consultations is not a reality for these men, and the justification is based on work routines, which do not allow them to be absent from their jobs. However, they reported that, when they feel weak, they try to ignore it, believing that the body will recover independently, because they are strong:

*I solve it by taking medicine, avoiding certain foods, trying to sleep well, not getting upset and not getting stressed. I don’t like looking for illnesses, so I avoid going to the* [health center]*, and I don’t even have time for that, I work a lot and, when I get* [home], *I just want to take a shower and go to bed.* (P21)

Representative words “eat” (X^2^=27.88), “sleep” (X^2^=27.33), “improve” (X^2^=26.66) and “well” (X^2^=27.48) reinforce the idea that good eating and resting habits are ways to promote health. The habit of resting after lunch at home or at work was reported by 33 (82.50%) participants, appearing as an essential practice for continuity of tasks. However, sleep problems were mentioned due to the occurrence of pain in the lower back, a symptom possibly associated with work routines in the field:

*I think eating is the main medicine, because if you don’t eat properly, you soon become weak and a lot of bad things* [diseases/ conditions] *start to appear*. (P27)

*I need to rest a little after lunch, even at work. If I’m at home, I also like to sleep in the afternoon, but nothing long* [...]. *I just can’t sleep when I have a lot of back pain* [low back pain] *and I can’t find a good position* [referring to body position when lying down]. (P11)

In cases of urgency or emergency, on the occasion of accidents or manifestation of debilitating symptoms, men seek formal services, whether at the health unit or at the public hospital in a nearby municipality. It has been shown that this search often occurs at the insistence of a family member or when they feel that their health is seriously threatened, fearing death:

*When I feel like I’m getting sick, I immediately run to talk to someone at the* [health] *center to get some medicine or even an appointment at the downtown hospital* [in the municipality of Santa Izabel do Pará], *when things are more serious.* (P7)

## DISCUSSION

In thematic axis “Knowledge that guides ways of dealing with health” (class 4), perceptions about being healthy point to two aspects: popular/traditional knowledge, strongly rooted in *Quilombola* communities and actively shared between generations; and biomedical knowledge, acquired through formal teaching, especially from health professionals who work in communities, articulating with traditional knowledge and shaping itself to translate *Quilombola* men’s understandings on how to act to stay or become healthy.

In this regard, traditional knowledge presents cultural conceptions demonstrated by the use of elements of nature in everyday practices. This knowledge is empirically related to health, given the need to solve daily problems, resulting in convictions and beliefs shared intergenerationally^(15)^.

*Quilombola* peoples have relevant knowledge about plants and their medicinal use, which have the property of contributing to generating beneficial reactions in the human body^(16)^. This was evidenced by the description of use of teas, leaves and herbs to treat illnesses and conditions, controlling signs and symptoms, suggesting that, even without knowing the active ingredients, men used plants to feel healthier. In view of this, phytotherapy is one of the first treatment measures used to solve health problems.

In turn, biomedical knowledge reflects a set of men’s experiences with health professionals^(17)^. In this context, following the creation of the Brazilian National Policy for Comprehensive Health of Rural and Forest Populations (PNSIPCF - *Política Nacional de Saúde Integral das Populações do Campo e da Floresta*), in 2011, rural communities began to have greater contact with the *Sistema Único de Saúde* (SUS - Brazilian Health System), strengthening access to services, especially through FHS, in daily care for rural populations’ individual and collective needs. It can be stated that health services in the countryside gradually allowed sociocultural conceptions about the health-disease process to be valued and integrated with technical-scientific convictions, favoring their coexistence^(10)^.

However, it is necessary to undertake governmental and administrative-operational efforts to guarantee these men’s access and retention in services, which still represents an important challenge for public health. From this perspective, it is highlighted that welcoming by professionals is essential to provide access to services and motivate men to seek them, especially in challenging scenarios, such as in *Quilombola* communities^(8)^.

Scientific literature shows that this population group underuses services and/or self-assesses assistance as poor or very poor^(18, 19)^. The opening hours of health units do not effectively meet the availability of users who live in these communities, especially men who, due to work activities, often choose not to dedicate a day or work shift to going to the unit, increasing the underutilization of services^(4)^.

In thematic axis “Being healthy as a synonym for the absence of disease and body-work” (classes 3 and 2), the dimension of recognizing oneself as healthy, in the context of class 3, is due to the absence of diseases or signs and symptoms. In participants’ imagination, the idea prevailed that the body is healthy if it does not manifest painful events. Thus, physical pain intensity is an indicator of illness or injury that can determine the body’s limits for daily tasks, interfering with daily eating, sleeping and work capacity.

For a long time, science and biomedical knowledge used this definition as true, understanding that health was limited to the absence of diseases or signs and symptoms^(19)^. However, following the evolution of knowledge about this imagination, studies question ideas that reduce man to needs that do not go beyond the physical body, as they exclude from the scope of considerations on the health-disease process social or individual factors, so-called “subjective”, which strongly implicate health interventions^(20, 21, 22)^.

It is understood that a social group’s knowledge is situated in a cultural context that can reveal several nuances in their socialization processes. It is essential to understand these processes to assess where ideas that are reproduced in that group come from. To this end, it is worth reflecting on the oppression and exclusion that historically characterize *Quilombola* communities^(23)^.

The consequences of a slavery regime are still noticeable today: poverty, violence and discrimination that affect black people appear as direct reflections of a country that institutionalized prejudice against this group, leaving them on the margins of society, denying access to basic rights, such as health and education^(^18,23-24^)^. This unfolds in the popular imagination, which reflects the domination of hegemonic thoughts or ideas, such as the biomedical model^(18, 23)^.

In turn, in class 2, the interpretation of being healthy is permeated by the intrinsic experience of men with their work activities. From this perspective, the idea of health occurs through the perception of physical body as an essential work tool, with the prevailing idea that routine and the work environment itself can contribute to maintaining health and well-being.

In men’s daily lives, it was identified that work translates into personal satisfaction, since, through it, achievements and achievements are obtained. According to Marxist thought, work appears as men’s interaction with the natural world, being the way in which they appropriate nature to meet their needs^(25)^.

The recognition of the importance between work and health was revealed in the attribution of meaning between being healthy and ability to perform tasks, understanding bodies as machines, linked to the idea of “body-work”. In this context, work takes over the function of dignifying man as a means to achieve material goods and obtain personal satisfaction. Corroborating this result, studies have shown that rural work allows men to transform nature into products that meet their needs and that, through it, human beings acquire and develop new skills as they carry it out^(25, 26)^.

In thematic axis “Importance of the home and family environment in the health context” (class 1), it was evident that men’s care practices develop in the contexts of family nucleus and domestic environment. The family nucleus corresponds to family members who co-participate in care actions as a support network or informal social support.

This network can offer different types of care, constituting a relevant factor in preventing vulnerabilities and social isolation. Generally, support is offered by those with whom bonds are maintained^(^17,19^)^, such as family, friends, neighbors and co-workers, with the family being the main support in caring for *Quilombola* men, especially in cases of illness and greater clinical complexity^(27)^.

In these men’s routine, social support networks are included in collective care in the family context to avoid risk situations, preventing injuries and promoting the family’s joint health. Thus, the adoption of preventive practices by men can be reinforced by their support networks, favoring the socialization of male needs and reducing personal barriers that limit health care^(2)^.

The female figure stood out in preventive and health promotion practices for men, which transfer responsibility for caring for them when they are ill to women. In this scenario, they are directly and often exclusively responsible for caring for others. Historically, the division of tasks between genders placed women in the position of home and family keeper, and their tasks were focused on caring for children, sick individuals and older adults^(17, 27)^.

Thus, in participants’ imagination, their home is characterized as a supporting and motivating agent in the search for being healthy. They interpret that this environment is part of their identity construction as men in the field, and, at the same time, it is consistent with their health condition and the access they have to individual and collective hygiene conditions.

Strongly engendered in common sense, the concepts about healthy housing, revealed in this study, are in line with evidence about risks in homes’ internal and external environments, which can compromise human and environmental health^(28)^. Thus, the relationship between health conditions and health construction in the environmental context is solidified, attesting to the relevance of the implications of the domestic environment in the health-disease process^(28)^.

Finally, thematic axis “Practices to prevent and solve health problems” (class 5) discusses that, in general, men seek to solve their problems through self-medicine to control signs and symptoms. There is no care routine with regular monitoring by FHS professionals, nor the immediate search for health services when sick. The justification for low demand for services is the incompatibility between their work routines and health services’ availability.

It was found that this practice is common among men, prioritizing occupational activities to the detriment of self-care and the search for care units. This fact finds meaning in the social construction of masculinity, which influences their decision to seek services or not, since there is a tendency to prioritize work, considering it as a fundamental element to maintain their historical role as providers of their homes^(25, 27)^.

Practices also alluded to good nutrition and quality of sleep. According to participants’ view, good nutrition is characterized by intake of natural foods, such as fruits and vegetables instead of high consumption of salt and fats. A study carried out in a *Quilombola* community in Japaratuba, state of Sergipe, identified similar results, showing that care with food occurred due to low intake of salt, fats and sugars, and frequent intake of fruits, vegetables and greens^(29)^.

For them, it is essential to have good quality sleep and rest, in order to maintain or obtain well-being and renew energy to carry out daily activities. This common sense knowledge is in line with evidence from scientific literature, as the concept of healthy sleep considers a multidimensional pattern of waking up/waking adapted to individual, social and environmental demands, promoting physical and mental well-being. Changes in quality and duration can increase the risk of harmful effects, such as the occurrence of diseases^(30)^.

Concerning practices in more complex situations, they reported seeking services, but this only occurs when their health condition is highly serious, in emergency situations. The path they follow directly affects their health conditions, as delay in seeking care delays the diagnosis of diseases and illnesses, causing them to enter services with serious and sometimes irreversible complications.

This search constitutes a set of decisions that they adopt to obtain health care. Thus, the different resources accessed and the trajectories in the search for care demarcate a therapeutic itinerary, which is not only characterized by choices centered on subjects, but also on the support networks and interpersonal relationships that surround them. Furthermore, therapeutic itinerary should not be understood solely as the linear description of individual choices regarding institutional health resources, since it relates to a variety of knowledge inherent to subjects’ sociocultural reality, which allows them to construct concepts and values to deal with their health-illness-care processes^(31)^.

Focusing on advancing *Quilombola* men’s health conditions, it is understood that social determinant analysis makes it possible to understand the details that make up their reality, both in the cultural aspect, with traditional practices related to gender issues, and in the understanding of their subjectivities, in the different elements of social life, mainly their work routines and the importance they attribute to them^(32)^. This reflection is made considering that occupational activities commonly interfere with the search for care and continuity of effective care, as demonstrated in this study.

It is wor th highlighting that formal care, provided by health professionals, must be strongly based on SUS universality, completeness and equity, doctrinal principles. Therefore, joint efforts are necessary between the subject, their family, their community, professionals and managers, strengthening support links, promoting and facilitating access to services. Therefore, co-participation of these actors is fundamental, as it encourages men to seek and remain in services, contributing to developing more appropriate health conditions for different human groups, such as *Quilombola* communities^(8, 10)^.

The evidence presented here portrays the local reality of the production of knowledge and practices necessary to understand how *Quilombola* men’s health develops in regional and national contexts, with theoretical-reflexive implications that make it possible to discuss the topic on the international scene. Therefore, recognizing and valuing the elements that involve these men’s knowledge and practices is fundamental to promoting self-care as a set of attitudes and behaviors that make it possible to maintain, modify, recover and manage health conditions with autonomy; promote effective multidisciplinary care; and debate/articulate public policies aimed at the biopsychosocial needs of this public, considering the historical, sociocultural, ethnic and political complexity that involves their way of life based on universal, comprehensive, equitable and prejudice-free care^(8, 19, 28, 29)^.

On a daily basis, *Quilombola* men experience and share knowledge and practices inherited from their ancestors, permeated by symbolic elements and material elements with cultural significance that differentiate them from other rural groups. From this perspective, it is worth highlighting the important role of health professionals, responsible for implementing strategies foreseen in policies that serve these communities and establishing new possibilities of care. This understands the importance of encouraging practices to reconfigure limiting knowledge, promoting greater health and autonomy to *Quilombola* men in their daily lives^(8, 24, 31)^.

### Study limitations

Carrying out the study in two *Quilombola* communities in a municipality in Pará, as they constitute a geographically specific setting, may limit, at least partially, the generalization of results as well as their interpretations and discussions in other contexts of men’s health care, such as those who live in urban areas, taking into account the local and regional particularities of this scenario, characterized by sociocultural, political-economic, administrative and operational aspects.

### Contributions to nursing, health, or public policies

In the fields of nursing and health, it is understood that the study can encourage the development of critical-reflective activities in care, management, teaching and research to strengthen government actions and social actors involved in health care of this public, especially in PHC, the scenario in which it was carried out, but which can also extend to secondary/specialized care and tertiary/hospital care.

In the case of PHC, evidence can favor ongoing education processes, resulting in the training of committed and empowered multidisciplinary teams based on knowledge about the peculiarities of this population group. Through the attributes of primary care, this makes it possible to address barriers and inequities in access to health actions and services, reaffirming the commitment to meeting biopsychosocial needs.

Additionally, the study can support discussions that make it possible to develop or reformulate care strategies whose implementation and/or implementation culminate(s) in advancing *Quilombola* men’s health conditions and transforming, to some degree, the challenging social reality in which they live.

## FINAL CONSIDERATIONS

*Quilombola* men’s knowledge about health revealed aspects that highlight the popular/traditional wisdom culturally apprehended and permeated by biomedical knowledge, shaping itself to translate their understandings about how to act to remain or become healthy. The interpretation of health as the absence of disease still permeates the understanding of this group, and the understanding of being healthy strongly considers working capacity as the main indicator of health, focusing on the physical body as a work instrument and revealing the importance of work in the process of dignity and personal satisfaction.

In care practices, their intimate relationship with their domestic and family environment proved to be essential for maintaining health, as this scenario serves as personal motivation to take care of themselves. Furthermore, they generally share these practices with a female figure, revealing that the act of caring is still linked to gender roles.

It was evidenced that the reproduction of masculinity as a hegemonic model contributes to building knowledge and practices in the context of health, since decisions related to it are based on sexist imagination that brings the feeling of being strong, healthy and less susceptible to diseases, limiting the search for care and potentially compromising well-being. Thus, there is a close relationship between gender issues, cultural roots and self-care patterns, which is why men have demonstrated that they prioritize work routines instead of self-care, considering that this behavior would bring personal fulfillment and better health conditions, not recognizing that health promotion and illness prevention strategies are also fundamental.

The results of this study point to the need to discuss and strengthen public policies aimed at men’s health in different geographic contexts, corroborating the importance of making efforts to value and better understand the topic, with the aim of rethinking multidisciplinary care practices, especially in sociocultural scope of *Quilombola* men. Therefore, it is necessary to invest in the development of other research that helps to understand aspects not covered here. Furthermore, it is important that managers and health teams understand the nuances of masculinity and local culture, as, together, they contribute to the absence of men in health services.
